# Author Correction: An actin filament branching surveillance system regulates cell cycle progression, cytokinesis and primary ciliogenesis

**DOI:** 10.1038/s41467-024-48240-1

**Published:** 2024-05-13

**Authors:** Muqing Cao, Xiaoxiao Zou, Chaoyi Li, Zaisheng Lin, Ni Wang, Zhongju Zou, Youqiong Ye, Joachim Seemann, Beth Levine, Zaiming Tang, Qing Zhong

**Affiliations:** 1 Key Laboratory of Cell Differentiation and Apoptosis of Chinese Ministry of Education, Department of Pathophysiology, Shanghai Jiao Tong University School of Medicine (SJTU-SM), Shanghai, China; 2 Center for Autophagy Research, Department of Internal Medicine, University of Texas Southwestern Medical Center, Dallas, TX USA; 3 Shanghai Institute of Immunology, Department of Immunology and Microbiology, Shanghai Jiao Tong University School of Medicine, Shanghai, China; 4 Department of Cell Biology, University of Texas Southwestern Medical Center, Dallas, TX USA; 5 Howard Hughes Medical Institute, University of Texas Southwestern Medical Center, Dallas, TX USA

**Keywords:** Centrosome, Actin, Cancer

Correction to: *Nature Communications* 10.1038/s41467-023-37340-z, published online 27 March 2023

The original version of this Article contained an error in the Methods section, in the subsection labelled Pyrene-actin polymerization assay, which incorrectly omitted details of how data was processed. The following sentence has been added: “The data has been processed as follows: the raw data has been normalized so that the initial background fluorescence values for all experimental conditions are roughly the same, and this data has then been fitted into smooth curves using the Graphpad Prism function ‘Nonlin fit:log(agonist) vs. response – Variable slope’”. This has been corrected in both the PDF and HTML versions of the Article. In addition, the original version of the Source Data file associated with this Article omitted raw unprocessed data for Figs. 1d, 1i, S1d, S1e, S1k, and S1l. The HTML has been updated to include a corrected version of the Source Data file.

### Supplementary information

Updated Source Data

