# Supplementary material for: An actin filament branching surveillance system regulates cell cycle progression, cytokinesis and primary ciliogenesis
Source: Nat Commun. 2023 Mar 27;14:1687. doi: 10.1038/s41467-023-37340-z (PMC10042869; doi:10.1038/s41467-023-37340-z)
Supplement: Supplementary file 1 — Supplementary Information [file 41467_2023_37340_MOESM1_ESM.pdf]

Supplementary Information

An Actin Filament Branching Surveillance System Regulates Cell Cycle Progression, Cytokinesis and Primary Ciliogenesis

Muqing Cao[1+*], Xiaoxiao Zou[1+], Chaoyi Li[1+], Zaisheng Lin[1], Ni Wang[1], Zhongju Zou[2], Youqiong Ye[3], Joachim Seemann[4], Beth Levine[2, 5], Zaiming Tang[1*], and Qing Zhong[1*]

[1]Key Laboratory of Cell Differentiation and Apoptosis of Chinese Ministry of Education, Department of Pathophysiology, Shanghai Jiao Tong University School of Medicine (SJTU-SM), Shanghai 200025, China.

[2]Center for Autophagy Research, Department of Internal Medicine, University of Texas Southwestern Medical Center, Dallas, Texas 75390, USA.

[3]Shanghai Institute of Immunology, Department of Immunology and Microbiology, Shanghai Jiao Tong University School of Medicine, Shanghai 200025, China.

[4]Department of Cell Biology, University of Texas Southwestern Medical Center, Dallas, Texas 75390, USA.

[5]Howard Hughes Medical Institute, University of Texas Southwestern Medical Center, Dallas, Texas 75390, USA.

[+]These authors contributed equally to this study.

*To whom correspondence should be addressed.

E-mail: qingzhong@shsmu.edu.cn, Muqingcao@sjtu.edu.cn, zaimingtang2017@shsmu.edu.cn.

This PDF file includes:

Supplementary Figures 1 to 7

Supplementary Table 1

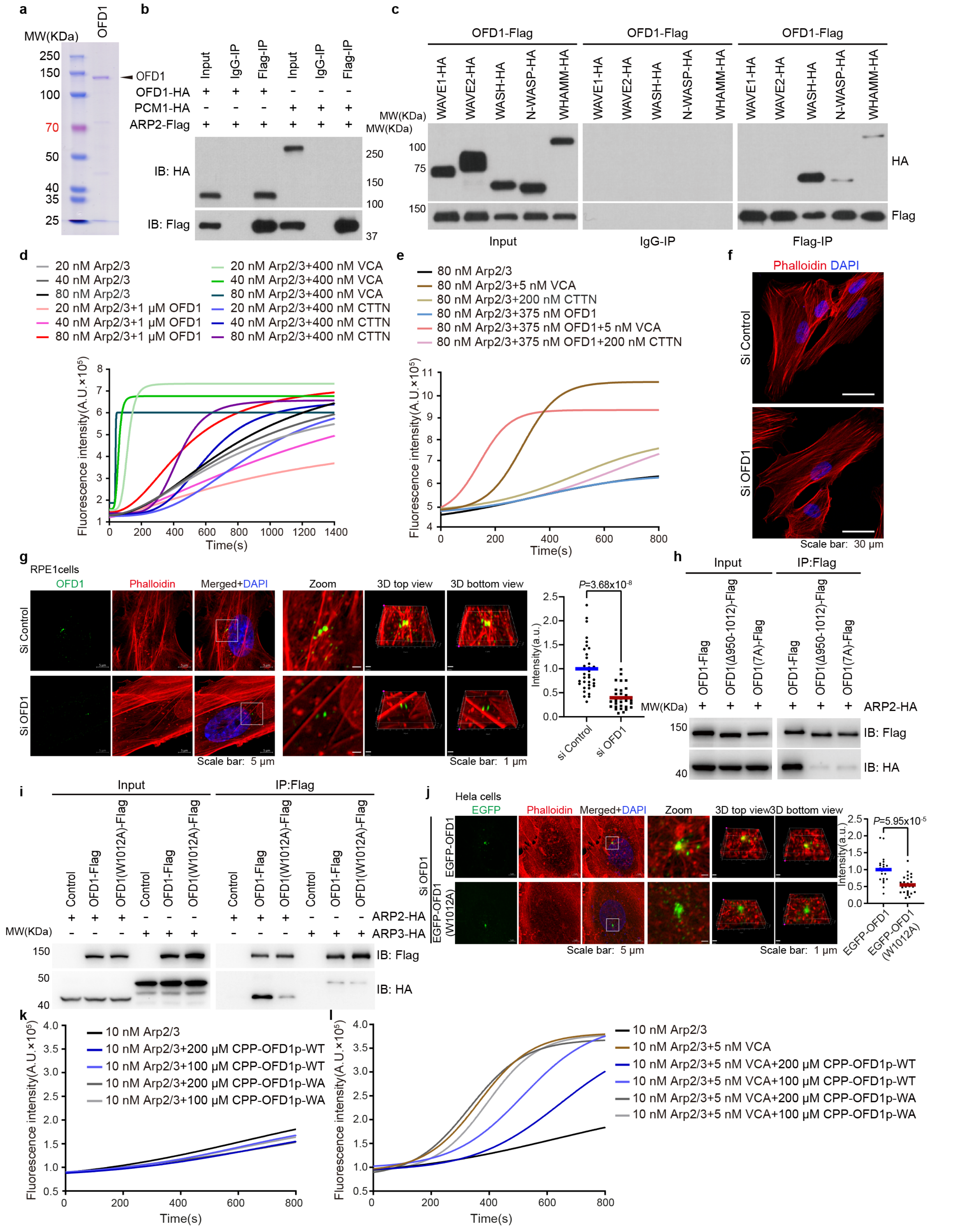

Supplementary Fig. 1. OFD1 functions as a class II NFP to promote actin filament branching. a

SDS-PAGE analysis of the purified recombinant human OFD1-Flag from EXPI 293S cells. **b** Immunoblot analysis of co-immunoprecipitation (IP) of ARP2-Flag with OFD1-HA or PCM1-HA. **c** Immunoblot analysis of the co-immunoprecipitation (IP) of OFD1 with WAVE1, WAVE2, WASH, N-WASP, and WHAMM in 293T cells. **d** Stimulation of actin polymerization by OFD1, GST-VCA, or cortactin (CTTN). Conditions were as follows: 0.875 μM 20% pyrene-labelled actin, 20 nM, 40 nM, 80 nM Arp2/3 complex, 1 μM OFD1, 400 nM GST-VCA, and 400 nM CTTN. **e** OFD1 has no synergy with CTTN on promoting actin branching. Polymerization of 0.875 μM 20% pyrene-labelled actin monomers was carried out in the presence of 5 nM GST-VCA or 200 nM cortactin or 375 nM OFD1, and 80 nM Arp2/3 complex. **f** Loss of OFD1 does not alter global F-actin network in RPE1 cells. Representative images of phalloidin staining of RPE1 cells to show the F-actin (red) network. **g** OFD1 promotes centrosomal actin branching. Representative imaging of endogenous OFD1 (green) and F-actin (phalloidin, red, Gamma-adjusted (0.5)) in RPE1 cells transiently transfected with control or OFD1 siRNA for 72 hours and after fixation with PFA-PEM (Left Panel). 3D reconstruction of the zoom images (step size: 140 nm) was performed using Imaris Viewer software. F-actin fluorescence integrated over a 3-μm-diameter circle around the centrosome for si-Control or si-OFD1 condition. Data shown represented as mean values ± SD, 35 si-Control cells and 31 si-OFD1 cells examined over three independent experiments, $P = 3.68\times10^{-8}$, two-tailed unpaired student's *t*-test (Right Panel). **h** Immunoblot analysis of co-immunoprecipitation (IP) of ARP2-HA with OFD1-Flag, OFD1(Δ950-1012)-Flag, or OFD1(7A)-Flag in 293T cells. OFD1(7A), OFD1(D996A, E998A, E1006A, E1007A, D1009A, D1010A, W1012A). **i** Immunoblot analysis of co-immunoprecipitation (IP) of ARP2-HA with Flag-vector, OFD1-Flag, or OFD1(W1012A)-Flag; and ARP3-HA with Flag-vector, OFD1-Flag, or OFD1(W1012A)-Flag. **j** Representative imaging of EGFP-OFD1 or EGFP-OFD1(W1012A) and F-actin (phalloidin, red, Gamma-adjusted (0.5)) in Tet-inducible EGFP-OFD1-expressing or EGFP-OFD1(W1012A)-expressing HeLa cells treated with 0.1 ng/mL Doxycycline and OFD1 siRNA for 72 hours and after fixation with PFA-PEM (Left Panel). 3D reconstruction of the zoom images (step size: 200 nm) was performed using Imaris Viewer software. F-actin fluorescence integrated over a 3-μm-diameter circle around the centrosome for EGFP-OFD1 or EGFP-OFD1(W1012A) condition. Data shown represented as mean values ± SD, 21 EGFP-OFD1-expression cells and 24 EGFP-OFD1-expression cells examined over three independent experiments, $P = 5.95\times10^{-5}$, two-tailed unpaired

student's *t*-test (Right Panel). **k** OFD1 peptides alone did not alter the actin polymerization rate. Polymerization of 0.875 μM 20% pyrene-labelled actin monomers was carried out in the presence of 10 nM Arp2/3 complex, 5 nM GST-VCA with 200 or 100 μM CPP-OFD1p-WT peptides, or with 200 or 100 μM CPP-OFD1p-W1012A (WA) peptides. **l** CPP-OFD1p-WT peptides inhibit the effect of class I NPF on promoting actin branching. Polymerization of 0.875 μM 20% pyrene-labelled actin monomers was carried out in the presence of 10 nM Arp2/3 complex with 200 or 100 μM CPP-OFD1p-WT peptides or with 200 or 100 μM CPP-OFD1p-W1012A (WA) peptides.

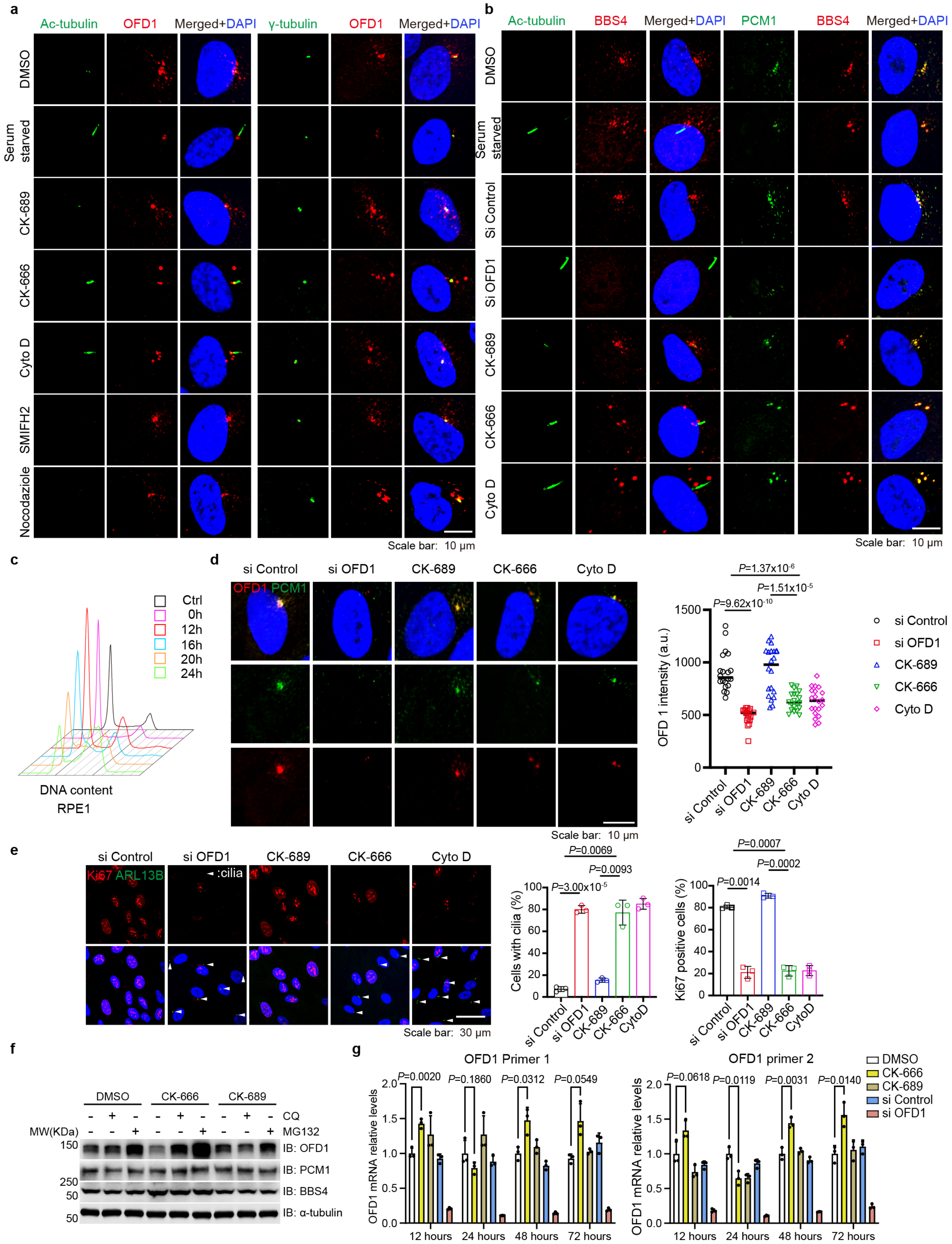

Supplementary Fig. 2. OFD1 is dynamically regulated upon actin filament reorganization. a

hTERT-RPE1 cells were co-stained with antibodies against OFD1 (red) and Ac-tubulin (green) or against OFD1 and γ-tubulin (green). Cells were subjected to DMSO, serum starvation, 120 μM CK-689, 120 μM CK-666, 100 nM Cyto D, 20 μM SMIFH2 treatment for 96 hours, si Control, si OFD1 treatment for 72 hours or 15 ng/mL nocodazole treatment for 48 hours. Scale bars: 10 μm. **b** hTERT-RPE1 cells were co-stained with antibodies against BBS4 (red) and Ac-tubulin (green) or against BBS4 and PCM1 (green) . Cells were subjected to DMSO, serum starvation, 120 μM CK-689, 120 μM CK-666, 100 nM Cyto D, or 20 μM SMIFH2 treatment for 96 hours, or indicated siRNA treatment for 72 hours. Scale bars: 10 μm. **c** hTERT-RPE1 cells were serum-starved for 72h to synchronize cells and released (time 0) for increasing time periods in fresh medium with serum as indicated. Cells were analyzed by flow cytometry to calculate the percentage of cells in each phases of the cell cycle. **d** Representative the SUM Z projection images of OFD1 (red) and PCM1 (green) staining in Synchronized hTERT-RPE1 treated with indicated siRNA, 120 μM CK-689, 120 μM CK-666 or 100nM Cyto D for 72h (Left Panel). Quantitative data of OFD1 intensity for the staining (Right Panel), Data shown represented as mean values ± SD, 200 cells examined over three independent experiments, $P = 9.62\times10^{-10}$, $P = 1.37\times10^{-6}$, $P = 1.51\times10^{-5}$, two-tailed unpaired student's *t*-test. **e** Representative images of Ki67 (red) and ARL13B (green) staining in Synchronized hTERT-RPE1 treated with indicated siRNA, 120 μM CK-689, 120 μM CK-666 or 100nM Cyto D for 72h. Cilia are marked by arrowheads (Left Panel). Quantitative data for the staining (Right Panel). Data shown represented as mean values mean ± SD, 300 cells examined over three independent experiments, $P = 3\times10^{-5}$, $P = 0.0069$, $P = 0.0093$, $P = 0.0014$, $P = 0.0007$, $P = 0.0002$, two-tailed unpaired student's *t*-test. **f** Immunoblot analysis of the protein levels of OFD1 and two other proteins known as satellite components, PCM1, and BBS4, were performed on samples from hTERT-RPE1 cells of indicated conditions. Cells were pretreated with DMSO, 120 μM CK-666, or 120 μM CK-689 for 96 hours before being treated with 20 μM CQ for 9 hours or 1 μM MG132 for 6 hours. **g** Relative mRNA expression levels of OFD1 normalized to GAPDH mRNA from hTERT-RPE1 cells treated with DMSO, 120 μM CK-666, 120 μM CK-689, or transfected with indicated siRNAs for 12 hours, 24 hours, 48 hours, or 72 hours. OFD1 mRNA levels were measured by real-time quantitative PCR in triplicate with two pairs of OFD1 primers. Data shown represented as mean values ± SD, error bar was defined as SD. $P = 0.002$, $P = 0.186$, $P = 0.031$, $P = 0.0549$, $P = 0.0618$, $P = 0.0119$, $P = 0.0031$, $P = 0.0140$, two-tailed unpaired student's *t*-test.

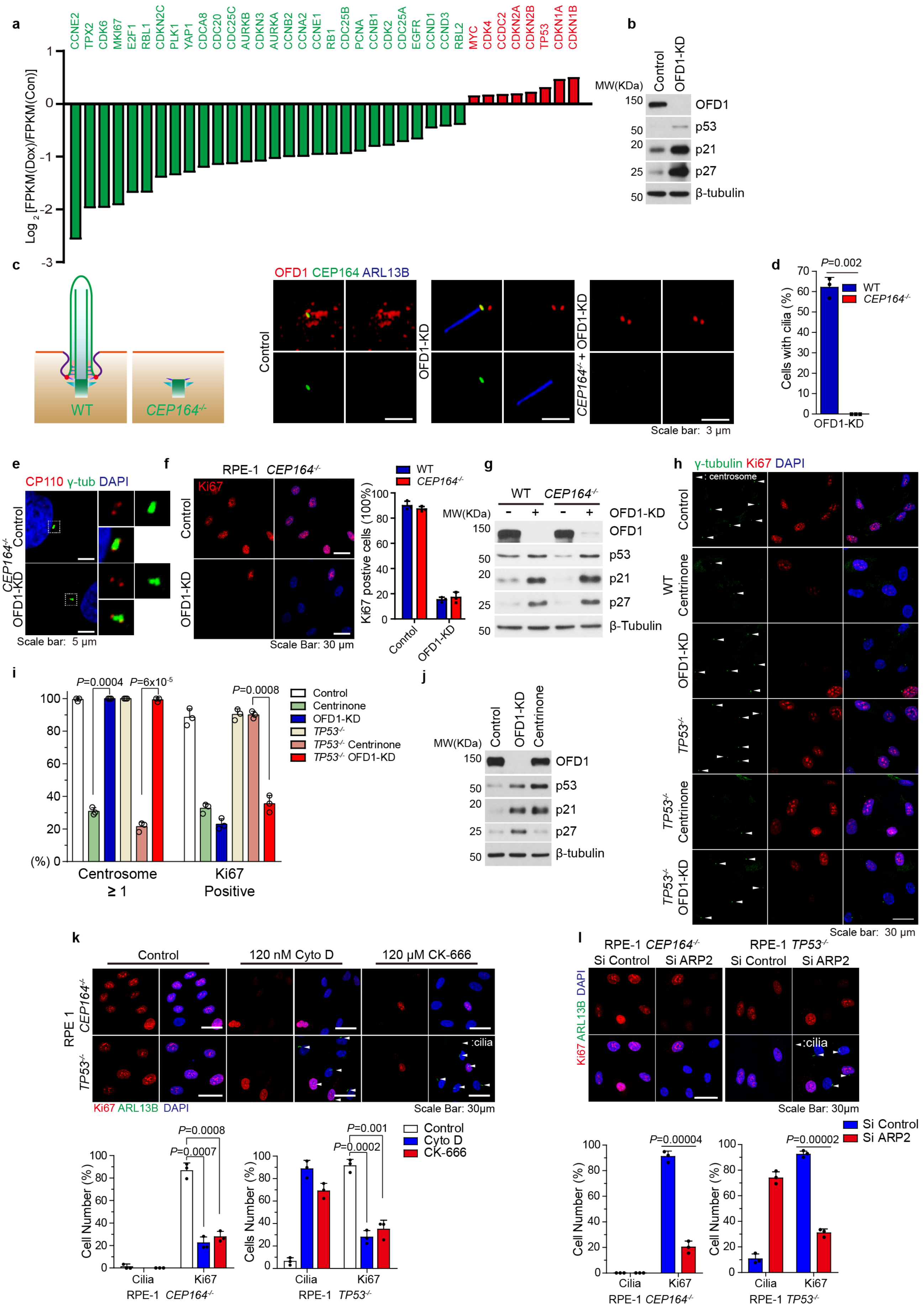
a
CCNE2 TPX2 CDK6 MKI67 E2F1 RBL1 CDKN2C PLK1 YAP1 CDCA8 CDC20 CDC25C AURKB CDKN3 AURKA CCNB2 CCNA2 CCNE1 RB1 CDC25B PCNA CCNB1 CDK2 CDC25A EGFR CCND1 CCND3 RBL2 MYC CDK4 CCDC2 CDKN2A CDKN2B TP53 CDKN1A CDKN1B
Log 2 [FPKM(Dox)/FPKM(Con)]
b
MW(KDa)
Control
OFD1-KD
OFD1
p53
p21
p27
β-tubulin
c
WT
CEP164-/-
OFD1 CEP164 ARL13B
Control
OFD1-KD
CEP164-/- + OFD1-KD
Scale bar: 3 μm
d
P=0.002
Cells with cilia (%)
WT
CEP164-/-
OFD1-KD
e
CP110 γ-tub DAPI
CEP164-/-
Control
OFD1-KD
Scale bar: 5 μm
f
RPE-1 CEP164-/-
Ki67
Control
OFD1-KD
Scale Bar: 30 μm
Ki67 postive cells (100%)
WT
CEP164-/-
Contol
OFD1-KD
g
WT
CEP164-/-
MW(KDa)
OFD1-KD
OFD1
p53
p21
p27
β-Tubulin
h
γ-tubulin Ki67 DAPI
◄: centrosome
Control
WT Centrinone
OFD1-KD
TP53-/-
TP53-/- Centrinone
TP53-/- OFD1-KD
Scale bar: 30 μm
i
P=0.0004
P=6x10-5
P=0.0008
Control
Centrinone
OFD1-KD
TP53-/-
TP53-/- Centrinone
TP53-/- OFD1-KD
(%)
Centrosome ≥ 1
Ki67 Positive
j
MW(KDa)
Control
OFD1-KD
Centrinone
OFD1
p53
p21
p27
β-tubulin
k
Control
120 nM Cyto D
120 μM CK-666
RPE 1
CEP164-/-
TP53-/-
:cilia
Ki67 ARL13B DAPI
Scale Bar: 30μm
P=0.0008
P=0.0007
Cell Number (%)
Cilia
Ki67
RPE-1 CEP164-/-
P=0.001
P=0.0002
Control
Cyto D
CK-666
Cells Number (%)
RPE-1 TP53-/-
l
RPE-1 CEP164-/-
RPE-1 TP53-/-
Si Control
Si ARP2
Ki67 ARL13B DAPI
:cilia
Scale Bar: 30μm
Si Control
Si ARP2
P=0.00004
P=0.00002
Cell Number (%)
Cilia
Ki67
RPE-1 CEP164-/-
RPE-1 TP53-/-

Supplementary Fig. 3. OFD1 ablation phenocopies actin filament debranching in forcing cycling non-transformed cells into quiescence. a Bar chart comparing gene expression from experiment in **3c**. **b** Immunoblot analysis of indicated proteins related to cell cycle progression. **c-f** Knockdown of OFD1 by RNAi in *CEP164*$^{-/-}$ RPE1 cells decreased Ki67-positive cells with no cilia formation. **c** Representative images of OFD1 (red), ARL13B (blue) and CEP164 (green)in WT, and *CEP164*$^{-/-}$ RPE1 cells with or without OFD1 knockdown. **d** Knockdown of OFD1 by RNAi in *CEP164*$^{-/-}$ RPE1 cells decreased cilia formation. 300 cells examined over three independent experiments, $P = 0.002$, two-tailed unpaired student's *t*-test. **e** Representative images of CP110 (red), γ-tubulin (green), and DAPI (blue) staining of CEP164 knockout RPE1 cells with or without OFD1 knockdown. **f** Knockdown of OFD1 by RNAi in *CEP164*$^{-/-}$ RPE1 cells decreased Ki67-positive (red) cells. 300 cells examined over three independent experiments, $P = 0.0004$, $P = 6\times10^{-5}$, $P = 0.0008$ two-tailed unpaired student's *t*-test. **g** Immunoblot analysis of indicated proteins from WT or *CEP164*$^{-/-}$ RPE1 cell lines with or without OFD1 knockdown. **h** Representative images of γ-tubulin (green) and Ki67 staining (red) of WT and OFD1 knockdown RPE1 cells with indicated genotype background. Cells were subjected to DMSO or 125 nM centrinone treatment for 72 hours. Cilia are marked by arrowheads. **i** Quantification of cells with positive γ-tubulin (green) and Ki67 (red) staining in **h**. 300 cells examined over three independent experiments, $P = 0.0004$, $P = 6\times10^{-5}$, $P = 0.0008$, two-tailed unpaired student's *t*-test. **j** Cell lysates of RPE1 cells treated as indicated were immunoblotted for OFD1, p53, p21, p27, and β-tubulin. **k** Representative images of ARL13B (green) and Ki67 (red) staining in *TP53*$^{-/-}$ or *CEP164*$^{-/-}$ RPE1 cells with indicated treatment for 48 hours (Upper Panel). 300 cells examined over three independent experiments, $P = 0.0007$, $P = 0.0008$, $P = 0.0002$, $P = 0.0001$, two-tailed unpaired student's *t*-test (Lower Panel). **l** Representative images of ARL13B (green) and Ki67 (red) staining of *TP53*$^{-/-}$ or *CEP164*$^{-/-}$ RPE1 cells transfected with control siRNA or ARP2 siRNA for 72 hours. Cilia are marked by arrowheads (Upper Panel). Quantitation of cells positive for ARL13B or Ki67 staining (Lower Panel). 300 cells examined over three independent experiments, $P = 0.00004$, $P = 0.00002$, two-tailed unpaired student's *t*-test (Lower Panel). All data shown represented as mean values mean ± SD, error bar was defined as SD.

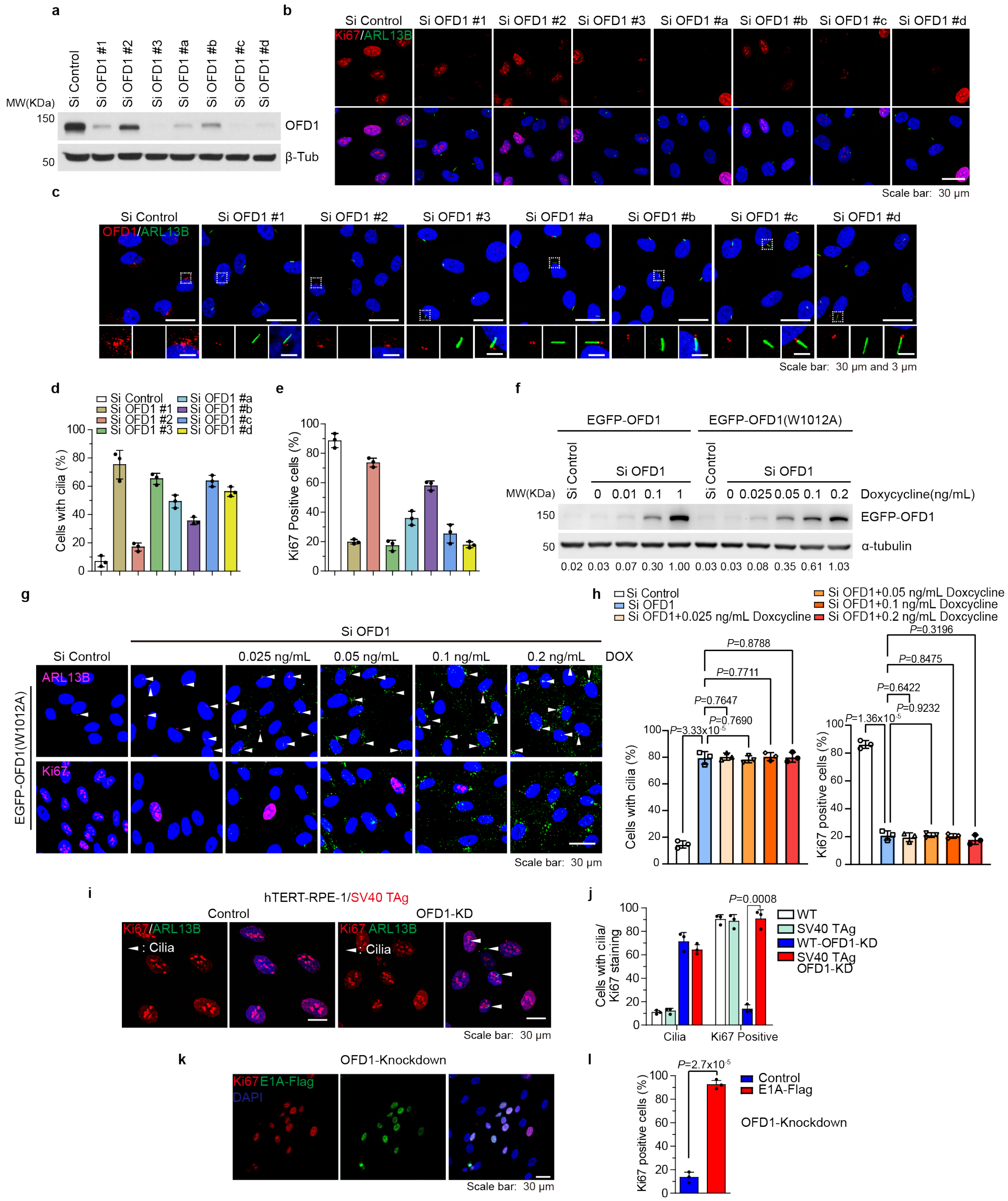

Supplementary Fig. 4. OFD1 ablation induced cell cycle arrest is dependent on the RB pathway in transformed cells. a-e Multiple siRNAs targeting OFD1 show similar effects on cilia formation and cell cycle arrest. **a** Immunoblot analysis for OFD1 and β-tubulin on RPE1 cell lysates transfected with indicated siRNAs. **b** Representative images of Ki67 (red) and ARL13B (green) staining of RPE1

cells transfected with indicated siRNAs. **c** Representative images of OFD1 (red) and ARL13B (green) staining of RPE1 cells transfected with indicated siRNAs. **d, e** Quantitation of cells positive for ARL13B or Ki67 staining. Data shown represented as mean values mean ± SD, error bar was defined as SD. 300 cells examined over three independent experiments, two-tailed unpaired student's *t*-test. **f** Immunoblot analysis for GFP and α-tubulin of Tet-inducible EGFP-OFD1 or EGFP-OFD1-W1012A expressing RPE1 cells with indicated titration of Doxycycline and indicated siRNAs. **g** Representative images of ARL13B and Ki67 staining in Tet-inducible EGFP-OFD1-W1012A-expressing RPE1 cells with indicated titration of Doxycycline and indicated siRNAs for 72 hours. Cilia are marked by arrowheads. **h** Quantitation of cells positive for ARL13B (red) or Ki67 (red) staining in **g**. Data shown represented as mean values mean ± SD, error bar was defined as SD. 300 cells examined over three independent experiments, $P = 3.33\times10^{-5}$, $P = 0.7647$, $P = 0.7690$, $P = 0.7711$, $P = 0.8788$, $P = 1.36\times10^{-5}$, $P = 0.6422$, $P = 0.9232$, $P = 0.8475$, $P = 0.3196$, two-tailed unpaired student's *t*-test. **i** Expression of SV40 T Antigen abolishes OFD1 loss-induced cell cycle arrest but not cilia formation. Representative immunofluorescence images of RPE1 cells stained for ARL13B (green) and Ki67 (red). Cilia are marked by arrowheads. Data shown represent mean ± SD. **j** Percentage of cells positive for ARL13B (red) or Ki67 (green) are from triplicate samples. Data shown represented as mean values mean ± SD, error bar was defined as SD. 300 cells examined over three independent experiments, P =0.0008, two-tailed unpaired student's *t*-test. **k** Representative images of Ki67 (red) and E1A-Flag (green) staining of OFD1 knockdown RPE1 cells. **l** Quantitation of cells positive for Ki67 staining. Data shown represented as mean values mean ± SD, error bar was defined as SD. 300 cells examined over three independent experiments, $P = 2.7\times10^{-5}$, two-tailed unpaired student's *t*-test.

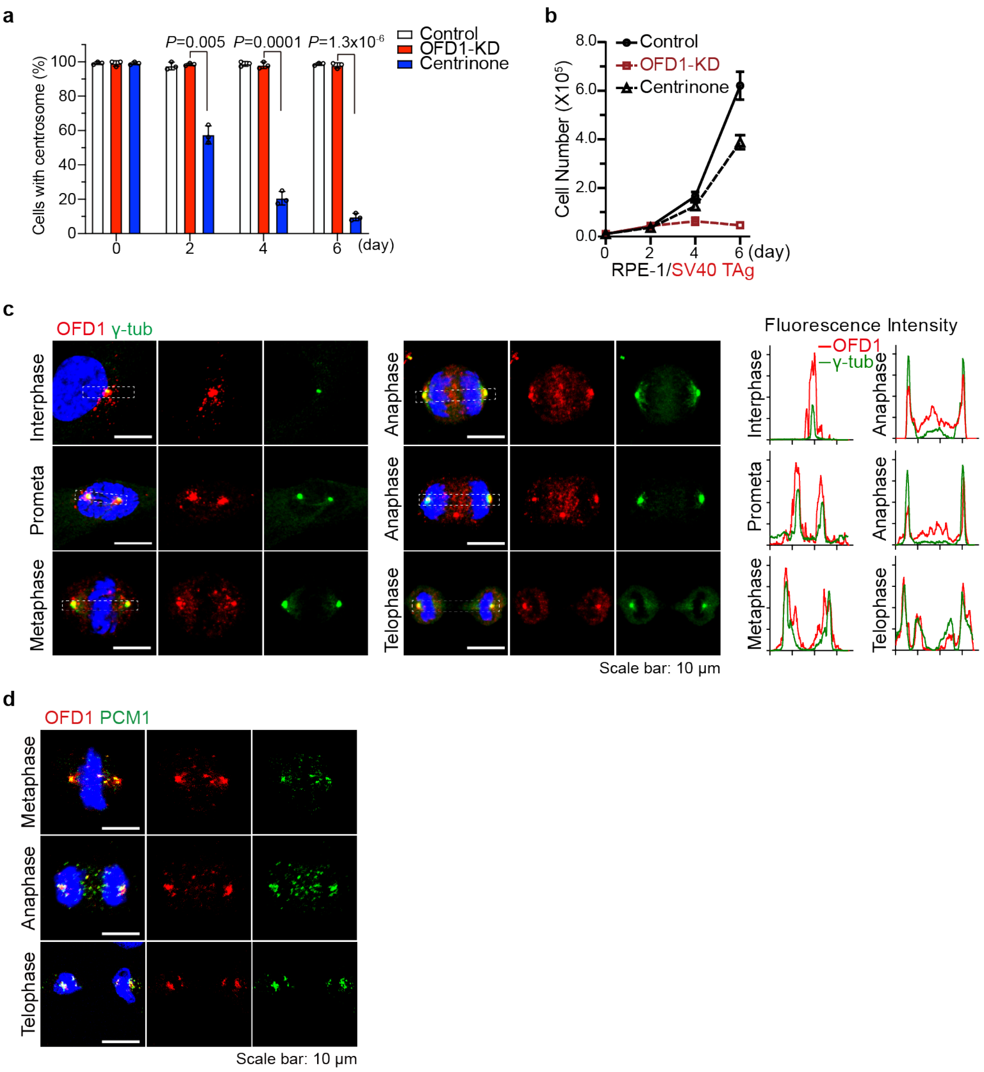

Supplementary Fig. 5. OFD1 localization and centrosome duplication during mitosis. a Quantitation of the percentage of cells with centrosomes. Data shown represented as mean values mean ± SD, error bar was defined as SD. 300 cells examined over three independent experiments, P = 0.005, P = 0.0001, P = 1.3×10^{-6}, two-tailed unpaired student's *t*-test. **b** Proliferation curves of RPE1/TAg cells with or without centrinone treatment. 300 cells examined over three independent experiments, data shown represented as mean values mean ± SD, error bar was defined as SD. **c** Representative images of OFD1 (red) and γ-tubulin (green) localization in the mitotic cell cycle of RPE1 cells (Left Panel). Scale bar, 10 μm. Fluorescence profile plots are indicated in the white-dotted line boxes (Right Panel). **d** Representative images of OFD1 (red) and PCM1 (green) localization in the mitotic cell cycle of RPE1 cells. Scale bar, 10 μm.

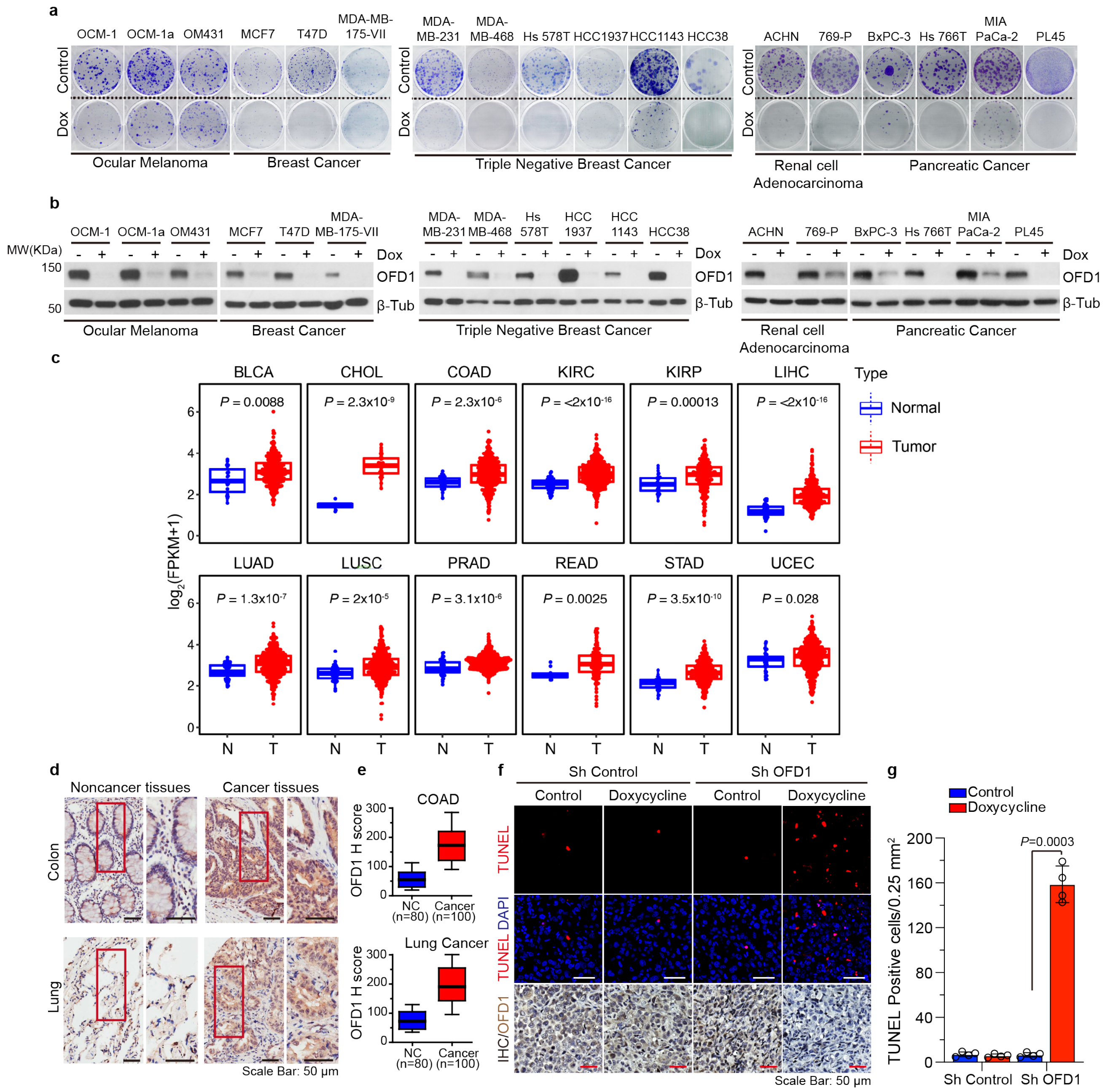

Supplementary Fig. 6. OFD1 sustains cancer cell proliferation and tumor growth. a Proliferation assay of indicated cell lines infected with a Doxycycline-inducible lentivirus encoding OFD1 shRNA in the presence or absence of Doxycycline. Cells were visualized by crystal violet staining. **b** Immunoblot analysis of OFD1 and β-tubulin from the cell lines in Supplementary Fig 6**a**. **c** Data summary of OFD1 mRNA levels normalized from fragments per kilobase of exon model per million reads mapped (FPKM) of tumor samples and adjacent normal samples obtained from The Cancer Genome Atlas (TCGA). Wilcoxon signed-rank test assesses the different expressions between tumor tissues and adjacent normal tissues. All box plots indicate the inter-quartile range (IQR), the middle line corresponds to the median, and the upper and lower whiskers represent observations within 1.5*IQR

(Q3 + 1.5*IQR or Q1 − 1.5*IQR), two-tailed unpaired student's *t*-test. **d** Immunohistochemistry labeling of OFD1 in tissue. **e** IHC scores for OFD1 staining in indicated cancer samples. The horizontal lines in the box plots represent the median, the boxes represent the interquartile range, and the whiskers represent the 10th and 90th percentiles. NC, non-cancer. Data shown represented as mean values mean ± SD, error bar was defined as SD. **f** Representative images of TUNEL-labeling and OFD1 staining of indicated PANC-1 xenograft tumor genotype in the absence or presence of Doxycycline in the animal diet. **g** Quantitation of TUNEL-positive (red) nuclei in indicated PANC-1 xenograft tumor genotype in the absence or presence of Doxycycline in the animal diet. Data shown represent mean ± SD, error bar was defined as SD. 300 cells examined over three independent experiments, P = 0.0003, two-tailed unpaired student's *t*-test.

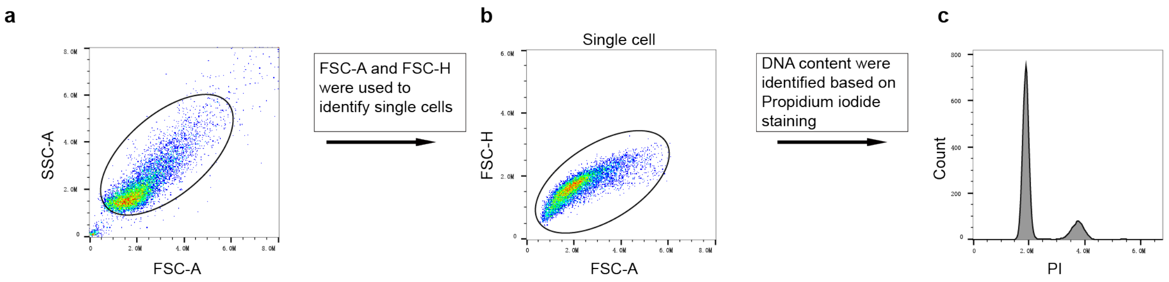

Supplementary Fig. 7. Schematic representation of FACS sequential gating/sorting strategies. **a** FSC-A vs. SSC-A gating was used to identify cells of interest based on size and granularity. **b** FSC-A vs. FSC-H density plot was used to exclude doublets. **c** Cells within the gate defined in **b** are represented in a histogram to evaluate the relative PI positive population.

Supplementary Table1. Sequences used in this paper.

Sequences	SOURCE	IDENTIFIER
ShControl (sense)<br>5′-TGGTTTACATGTCGACTAA-3′	This paper	N/A
ShOFD1 #3 (sense)<br>5′-GAACGAAGAGAACTAGAAA-3′	This paper	N/A
siRNA Non-targeting Control	Dharmacon	D-001810-10
siRNA ARP2 (sense)<br>5′-ACGGUUGGAACGAGAACUUAA-3′<br>5′-UUGGUGUGACUGUUCGAUAAA-3′	Sigma Aldrich	N/A
siRNA ARP3	Dharmacon	L-012077-00
siRNA ARPC2	Dharmacon	L-012081-00
siRNA JMY	Dharmacon	L-016922-00
siRNA N-WASP	Dharmacon	L-006444-00
siRNA OFD1	Dharmacon	L-009300-00
siRNA RB1	Dharmacon	L-003296-02
siRNA RBL1	Dharmacon	L-003298-00
siRNA RBL2	Dharmacon	L-003299-00
siRNA WAVE1	Dharmacon	L-011557-00
siRNA WAVE2	Dharmacon	L-012141-00
siRNA WASH	Dharmacon	L-190043-00
siRNA WHAMM	Dharmacon	L-022415-01
siRNA OFD1 #1 (sense)<br>5′-GAAUGAAGUGUACUGCAAU-3′	Sigma Aldrich	N/A
siRNA OFD1 #2 (sense)<br>5′- GAGACAGGAGCAGAAUAUA-3′	Sigma Aldrich	N/A
siRNA OFD1 #3 (sense)<br>5′-GAACGAAGAGAACUAGAAA-3′	Sigma Aldrich	N/A

siRNA OFD1 #a (sense)<br>5′-CAGACAAGUUCGACAUUUA-3′	Sigma Aldrich	N/A
siRNA OFD1 #b (sense)<br>5′-GACAAAGUCGAAAGUUUAA-3′	Sigma Aldrich	N/A
siRNA OFD1 #c (sense)<br>5′-GAUCGAUCGUUCUGUCAAU-3′	Sigma Aldrich	N/A
siRNA OFD1 #d (sense)<br>5′-CGAAAAGGCUAUAGUGGUU-3′	Sigma Aldrich	N/A
