# Supplementary material for: An actin filament branching surveillance system regulates cell cycle progression, cytokinesis and primary ciliogenesis
Source: Nat Commun. 2023 Mar 27;14:1687. doi: 10.1038/s41467-023-37340-z (PMC10042869; doi:10.1038/s41467-023-37340-z)
Supplement: Supplementary file 3 — Description of Additional Supplementary files [file 41467_2023_37340_MOESM3_ESM.pdf]

Description of Additional Supplementary files

File name: Supplementary Movie 1
Description: F-actin concentrates around the centrosomal region in control cells. RPE1 cells were transfected by control siRNA. OFD1 (Green, GFP-OFD1) and F-actin (Red, indicated by LifeAct-RFP).

File name: Supplementary Movie 2
Description: F-actin around the centrosomal region is less in OFD1 KD cells. RPE1 cells were transfected by siRNA targeting OFD1. OFD1 (Green, GFP-OFD1) and F-actin (Red, indicated by LifeAct-RFP).

File name: Supplementary Movie 3
Description: Live cell images of the fusion events of centrosomal OFD1 in Tetinducible EGFP-OFD1-expressing RPE1 cells. Cells were treated with 0.1 ng/mL Doxycycline and DMSO for 72 hours.

File name: Supplementary Movie 4
Description: Live cell images of the fission events of centrosomal OFD1 in Tetinducible EGFP-OFD1-expressing RPE1 cells. Cells were treated with 0.1 ng/mL Doxycycline and DMSO for 72 hours.

File name: Supplementary Movie 5
Description: Live cell images of the centrosomal OFD1 in Tet-inducible EGFPOFD1-expressing RPE1 cells treated with CK666. Cells were treated with 0.1 ng/mL Doxycycline and 120 µM CK-666 for 72 hours.

File name: Supplementary Movie 6
Description: Cell cycle progression of RPE1/TAg cells was monitored by time-lapse imaging. Cells were transfected by control siRNA.

File name: Supplementary Movie 7
Description: Cell cycle progression of RPE1/TAg cells with OFD1 depletion was monitored by time-lapse imaging. Cells were transfected by siRNA targeting OFD1.

File name: Supplementary Movie 8
Description: Cell cycle progression of RPE1/TAg cells was monitored by fluorescent time-lapse imaging. Cells were transfected by control siRNA. Microtubule (Green, GFP-tubulin) and F-actin (Red, indicated by LifeAct-RFP).

File name: Supplementary Movie 9
Description: Cell cycle progression of RPE1/TAg cells with OFD1 depletion was monitored by fluorescent time-lapse imaging. Cells were transfected by siRNA targeting OFD1. Microtubule (Green, GFP-tubulin) and F-actin (Red, indicated by LifeAct-RFP).
