# Supplementary material for: An actin filament branching surveillance system regulates cell cycle progression, cytokinesis and primary ciliogenesis
Source: Nat Commun. 2023 Mar 27;14:1687. doi: 10.1038/s41467-023-37340-z (PMC10042869; doi:10.1038/s41467-023-37340-z)
Supplement: Supplementary file 13 — Reporting Summary [file 41467_2023_37340_MOESM13_ESM.pdf]

nature portfolio

Corresponding author(s): Muqing Cao, Zaiming Tang, Qing Zhong

Last updated by author(s): Feb 16, 2023

Reporting Summary

Nature Portfolio wishes to improve the reproducibility of the work that we publish. This form provides structure for consistency and transparency in reporting. For further information on Nature Portfolio policies, see our Editorial Policies and the Editorial Policy Checklist.

Statistics

For all statistical analyses, confirm that the following items are present in the figure legend, table legend, main text, or Methods section.

n/a	Confirmed	
[ ]	[x]	The exact sample size (*n*) for each experimental group/condition, given as a discrete number and unit of measurement
[ ]	[x]	A statement on whether measurements were taken from distinct samples or whether the same sample was measured repeatedly
[ ]	[x]	The statistical test(s) used AND whether they are one- or two-sided<br>*Only common tests should be described solely by name; describe more complex techniques in the Methods section.*
[x]	[ ]	A description of all covariates tested
[x]	[ ]	A description of any assumptions or corrections, such as tests of normality and adjustment for multiple comparisons
[ ]	[x]	A full description of the statistical parameters including central tendency (e.g. means) or other basic estimates (e.g. regression coefficient) AND variation (e.g. standard deviation) or associated estimates of uncertainty (e.g. confidence intervals)
[ ]	[x]	For null hypothesis testing, the test statistic (e.g. *F*, *t*, *r*) with confidence intervals, effect sizes, degrees of freedom and *P* value noted<br>*Give P values as exact values whenever suitable.*
[x]	[ ]	For Bayesian analysis, information on the choice of priors and Markov chain Monte Carlo settings
[x]	[ ]	For hierarchical and complex designs, identification of the appropriate level for tests and full reporting of outcomes
[x]	[ ]	Estimates of effect sizes (e.g. Cohen's *d*, Pearson's *r*), indicating how they were calculated

Our web collection on statistics for biologists contains articles on many of the points above.

Software and code

Policy information about availability of computer code

Data collection	We used commercial software available with the respective instruments for data collection. Pyrene actin polymerization assay data and absorbance-based assay data were collected on an VICTOR Nivo fluorometer(Perkin Elmer). Westernblot data was collected on an Odyssey | machine and Image Studio software (LiCor) or Bio-Rad ChemiDoc Imaging System. Fluorescence images were taken by the FV3000 confocal microscope(Olympus Corporation),or IX83 microscope(Olympus Corporation), or LSM780, or LSM880 laser scanning confocal microscope equipped with an Airyscan module(Zeiss), or Delta Vision OMX SR microscope(GE).Other data collection methods were specified in the methods section describing each experimental method.
Data analysis	All softwares used to process and plot data in this study were listed in the methods section. Graphs and numerical data (including statistics/ error bars)were analyzed and plotted by Prism(GraphPad, Version 9.1.0); Image J(NIH, Version 1.53c)for the analysis of |microscopy data;FRAP experiments data was corrected using the Image J plug-in FRAP Profiler v2; Flow cytometry data was analyzed using FlowJo 10.8.1. Other data analysis methods were specified in the methods section describing each experimental method.

For manuscripts utilizing custom algorithms or software that are central to the research but not yet described in published literature, software must be made available to editors and reviewers. We strongly encourage code deposition in a community repository (e.g. GitHub). See the Nature Portfolio guidelines for submitting code & software for further information.

Data

Policy information about availability of data

All manuscripts must include a data availability statement. This statement should provide the following information, where applicable:

- Accession codes, unique identifiers, or web links for publicly available datasets
- A description of any restrictions on data availability
- For clinical datasets or third party data, please ensure that the statement adheres to our policy

All original data that support the findings of this study will be available upon request. Source data are provided with this paper. The raw data generated in this study including RNA-Seq have been deposited in the database of Gene Expression Omnibus (GEO) under accession code GSE225003. The individual-level data are available under restricted access for download, access can be obtained by authorized access only. The raw individual-level data are protected and are not available due to data privacy laws. The processed individual-level data are available at the database of Gene Expression Omnibus. The individual-level data generated in this study are provided in the Supplementary Information/Source Data file. The individual-level data used in this study are available in the GEO database under accession code GSE225003 [https://www.ncbi.nlm.nih.gov/geo/query/acc.cgi?acc=GSE225003].

Human research participants

Policy information about studies involving human research participants and Sex and Gender in Research.

Reporting on sex and gender	N/A
Population characteristics	N/A
Recruitment	N/A
Ethics oversight	N/A

Note that full information on the approval of the study protocol must also be provided in the manuscript.

Field-specific reporting

Please select the one below that is the best fit for your research. If you are not sure, read the appropriate sections before making your selection.

☒ Life sciences ☐ Behavioural & social sciences ☐ Ecological, evolutionary & environmental sciences

For a reference copy of the document with all sections, see nature.com/documents/nr-reporting-summary-flat.pdf

Life sciences study design

All studies must disclose on these points even when the disclosure is negative.

Sample size	Sample sizes were determined according to the related references (PMID: 11231575, PMID: 20230748, PMID: 35365629,PMID: 32376951) and chosen based on logistical and experimental experiences. In every case, three or two biologiclal replicates for each experiment were included. All experiments were reliably reproduced and the number of samples chosen was sufficient to support meaningful conclusions.
Data exclusions	In general, no data were excluded, except for experimental failures due to control failures and technical problems.
Replication	All microscopic, biochemical, and biological assays were independently repeated at least three times as mentioned in Methods or figure legends. All attempts at replication were successful.
Randomization	All groups were randomly assigned,with each group representing a different treatment or condition.
Blinding	Blinding was not applicable to this study as the data grouping and analysis were performed in an unbiased manner. Data was analyzed with common strategies.

Reporting for specific materials, systems and methods

We require information from authors about some types of materials, experimental systems and methods used in many studies. Here, indicate whether each material, system or method listed is relevant to your study. If you are not sure if a list item applies to your research, read the appropriate section before selecting a response.

Materials & experimental systems

n/a	Involved in the study
	x Antibodies
	x Eukaryotic cell lines
x	Palaeontology and archaeology
	x Animals and other organisms
	x Clinical data
x	Dual use research of concern

Methods

n/a	Involved in the study
x	ChIP-seq
	x Flow cytometry
x	MRI-based neuroimaging

Antibodies

Antibodies used

Bovine Anti-Goat Alexa Fluor 488, Jackson ImmunoResearch Laboratories, Cat#805-545-180, 1:500 dilution;
Goat Anti-Mouse Alexa Fluor 488, Jackson ImmunoResearch Laboratories, Cat#115-545-068, 1:500 dilution;
Goat Anti-Mouse Alexa Fluor 568, Life Technologies, Cat#A-11031, 1:300 dilution;
Goat Anti-Rabbit Alexa Fluor 594, Life Technologies, Cat#A-11012, 1:300 dilution;
Goat Anti-Rabbit Alexa Fluor 647, Life Technologies, Cat#A32733, 1:300 dilution;
Goat Anti-Mouse Alexa Fluor 647, Life Technologies, Cat# A32728, 1:300 dilution;
Goat anti-mouse IgG light chain specific HRP, Jackson ImmunoResearch Laboratories, Cat#115-035-174, 1:5000 dilution;
Goat polyclonal anti-CEP164, Santa Cruz, Cat#sc-240226, 1:500 dilution;
Mouse anti-rabbit IgG light chain specific HRP, Jackson ImmunoResearch Laboratories, Cat#211-032-171, 1:5000 dilution;
Mouse monoclonal anti-ARL13B, NeuroMab, Cat#75-287, Clone name: N295B/66, 1:1000 dilution;
Mouse monoclonal anti-β-tubulin, Developmental Studies Hybridoma Bank, Cat#E7, 1:10000 dilution;
Mouse monoclonal anti-Flag M2, Sigma Aldrich, Cat#F1804, Clone: M2, monoclonal, 1:3000 dilution (IF), 1:5000 dilution (WB);
Mouse monoclonal anti-GAPDH, Santa Cruz, Cat#sc-365062, 1:20000 dilution;
Mouse monoclonal anti-γ-tubulin, Sigma Aldrich, Cat#T6557, GTU-88, monoclonal, 1:2000 dilution;
Mouse monoclonal anti-HA, Sigma Aldrich, Cat#H9658, HA-7, monoclonal, 1:10000 dilution;
Mouse monoclonal anti-p21, Santa Cruz, Cat#sc-6246, 1:1000 dilution
Mouse monoclonal anti-PCM1, Santa Cruz, Cat#sc-398365, 1:1000 dilution;
Mouse monoclonal anti-p27 KIP1, Cell Signaling Technology, Cat#3698, 1:1000 dilution;
Mouse monoclonal anti-p53, Santa Cruz, Cat#sc-126, clone DO-1, 1:1000 dilution;
Mouse monoclonal anti-RB1, Cell Signaling Technology, Cat#9309, 1:1000 dilution;
Mouse monoclonal anti-α-tubulin, Simga Aldrich, Cat#T6199, 1:10000 dilution;
Mouse monoclonal anti-ARP2, Simga Aldrich, Cat#A6104, 1:200 dilution (IF), 1:2000 dilution (WB);
Rabbit polyclonal anti-ARL13B, Proteintech Group, Cat#17711-1-AP, 1:1000 dilution;
Rabbit polyclonal anti-IFT20, Proteintech Group, Cat#13615-1-AP, 1:1000 dilution;
Rabbit polyclonal anti-BBS4, Proteintech Group, Cat#12766-1-AP, 1:1000 dilution;
Rabbit polyclonal anti-Ki67, Abcam, Cat#ab15580, 1:500 dilution;
Rabbit polyclonal anti-GFP, Abcam, Cat# ab6556, 1:2000 dilution;
Rabbit polyclonal anti-RBL1, Proteintech Group, Cat#13354-1-AP, 1:500 dilution;
Rabbit polyclonal anti-RBL2, Proteintech Group, Cat#27251-1-AP, 1:600 dilution;
Rabbit polyclonal anti-OFD1, PMID: 33531668, 1:3000 dilution (IF), 1:5000 dilution (WB);
Rabbit polyclonal anti-CP110, Proteintech Group, Cat#12780-1-AP, 1:500 dilution;

Validation

All information and validation of commercial antibodies used in this work are available on the manufacturers' websites listed below:

Goat polyclonal anti-CEP164 (Santa Cruz, Cat#sc-240226) was validated in Weng, Rueyhung Roc et al. Biophysical journal vol. 115,2 (2018): 263-275. doi:10.1016/j.bpj.2018.04.051 and Loukil, Abdelhalim et al. The Journal of cell biology vol. 216,5 (2017): 1287-1300. doi:10.1083/jcb.201608119.

Mouse monoclonal anti-ARL13B, NeuroMab, Cat#75-287
https://www.antibodiesinc.com/products/anti-arl13b-antibody-n295b-66-75-287

Mouse monoclonal anti-β-tubulin, Developmental Studies Hybridoma Bank, Cat#E7
https://dshb.biology.uiowa.edu/E7_2

Mouse monoclonal anti-Flag M2, Sigma Aldrich, Cat#F1804
https://www.sigmaaldrich.cn/CN/zh/product/sigma/f1804

Mouse monoclonal anti-GAPDH, Santa Cruz, Cat#sc-365062
https://www.scbt.com/p/gapdh-antibody-g-9/

Mouse monoclonal anti-γ-tubulin, Sigma Aldrich, Cat#T6557
https://www.sigmaaldrich.cn/CN/zh/product/sigma/t6557

Mouse monoclonal anti-HA, Sigma Aldrich, Cat#H9658
https://www.sigmaaldrich.cn/CN/zh/product/sigma/h9658

Mouse monoclonal anti-p21, Santa Cruz, Cat#sc-6246
https://www.scbt.com/p/p21-antibody-f-5?requestFrom=search

Mouse monoclonal anti-PCM1, Santa Cruz, Cat#sc-398365
https://www.scbt.com/p/pcm1-antibody-g-6

Mouse monoclonal anti-p27 KIP1, Cell Signaling Technology, Cat#3698
https://www.cellsignal.com/products/primary-antibodies/p27-kip1-sx53g8-5-mouse-mab/3698

Mouse monoclonal anti-p53, Santa Cruz, Cat#sc-126
https://www.scbt.com/p/p53-antibody-do-1

Mouse monoclonal anti-Rb, Cell Signaling Technology, Cat#9309
https://www.cellsignal.com/products/primary-antibodies/rb-4h1-mouse-mab/9309

Mouse monoclonal anti-α-tubulin, Simga Aldrich, Cat#T6199
https://www.sigmaaldrich.cn/CN/zh/product/sigma/t6199

Mouse monoclonal anti-ARP2, Simga Aldrich, Cat#A6104
https://www.sigmaaldrich.cn/CN/zh/product/sigma/a6104

Rabbit polyclonal anti-ARL13B, Proteintech Group, Cat#17711-1-AP
https://www.ptglab.com/products/ARL13B-Antibody-17711-1-AP.htm

Rabbit polyclonal anti-IFT20, Proteintech Group, Cat#13615-1-AP
https://www.ptglab.com/products/IFT20-Antibody-13615-1-AP.htm

Rabbit polyclonal anti-BBS4, Proteintech Group, Cat#12766-1-AP
https://www.ptglab.com/products/BBS4-Antibody-12766-1-AP.htm

Rabbit polyclonal anti-Ki67, Abcam, Cat#ab15580
https://www.abcam.com/Ki67-antibody-ab15580.html

Rabbit polyclonal anti-GFP, Abcam, Cat# ab6556
https://www.abcam.com/gfp-antibody-ab6556.html

Rabbit polyclonal anti-RBL1, Proteintech Group, Cat#13354-1-AP
https://www.ptglab.com/products/RBL1-Antibody-13354-1-AP.htm

Rabbit polyclonal anti-RBL2, Proteintech Group, Cat#27251-1-AP
https://www.ptglab.com/products/RBL2-Antibody-27251-1-AP.htm

Rabbit polyclonal anti-OFD1, PMID: 33531668;

Rabbit polyclonal anti-CP110, Proteintech Group, Cat#12780-1-AP
https://www.ptglab.co.jp/Products/CP110-Antibody-12780-1-AP.htm

Eukaryotic cell lines

Policy information about cell lines and Sex and Gender in Research

Cell line source(s)	hTERT-RPE1, HeLa, ACHN, OCM-1, OCM-1a, OM431, MDA-MB-175-VII, hTERT-BJ1, IMR-90, Hs 578T, MCF7, MDA-MB-231, MDA-MB-468, T47D, HT-29, A549, Hs 766T, MIA PaCa2, PANC-1, PL45, HCC1937, HCC1143, HCC38, 769-P, BxPC-3, HEK293 cells were obtained from ATCC.
Authentication	The cell lines exhibited morphological features, growth characteristics and phenotypic responses consistent with original description of these cell lines. All cell lines were authenticated by ATCC STR testing.
Mycoplasma contamination	All cell lines used in experiments were tested negative for mycoplasma contamination.
Commonly misidentified lines (See ICLAC register)	No commonly misidentified cell lines were used in our study.

Animals and other research organisms

Policy information about studies involving animals; ARRIVE guidelines recommended for reporting animal research, and Sex and Gender in Research

Laboratory animals	6- to 8-week-old female NOD/SCID mice were used in this study. All mice were kept in group housing (3-5 mice per cage)in a specific pathogen-free facility with controlled environmental conditions of humidity (50±10%), lighting (a 12-h light/dark cycle)and controlled temperature (21±1°C) at the animal facility, UT Southwestern Medical Center (UTSW).
Wild animals	No wild animals were used in the study.
Reporting on sex	Female NOD/SCID mice were used in this study.
Field-collected samples	No field collected samples were used in the study.
Ethics oversight	All of the mouse experiments were performed according to the guidelines of the Institutional Animal Care and Use Committee (IACUC) at UTSW.

Note that full information on the approval of the study protocol must also be provided in the manuscript.

Clinical data

Policy information about clinical studies

All manuscripts should comply with the ICMJEguidelines for publication of clinical research and a completedCONSORT checklist must be included with all submissions.

Clinical trial registration	The tissue arrays contain tissues from 80 paired colon carcinoma and normal tissue samples together with extra 20 colon carcinoma samples (HColA180Su10-M-069), as well as 60 paired lung cancer and normal tissue samples (HLugC120PT01) were used to examine the expression profiles of OFD1 immunohistochemistry (IHC).
Study protocol	Colon cancer and lung cancer with corresponding normal tissue microarray (TMA) sections were prepared by Shanghai Outdo Biotech Co. Ltd. (Shanghai, China).
Data collection	The sample collection and preparation were approved by the Scientific Investigation Board of Taizhou Hospital and were in accordance with the ethical principles originating from the Declaration of Helsinki.
Outcomes	IHC stains were scored by two independent pathologists who were blinded to the clinical characteristics of the patients. The scoring system was based on the intensity and extent of staining: staining intensity was classified as 0 (negative), 1 (weak), 2 (moderate), or 3 (strong). Immunostained sections on microarrays were scored by multiplying the intensity (0-3) and area percentage (0-100%) of staining.

Flow Cytometry

Plots

Confirm that:

- [ ] The axis labels state the marker and fluorochrome used (e.g. CD4-FITC).
- [x] The axis scales are clearly visible. Include numbers along axes only for bottom left plot of group (a 'group' is an analysis of identical markers).
- [x] All plots are contour plots with outliers or pseudocolor plots.
- [ ] A numerical value for number of cells or percentage (with statistics) is provided.

Methodology

Sample preparation	RPE1 cells were harvested and washed with chilled PBS, and then fixed with 75% ethanol at -20°C overnight. The fixed cells were washed two times with PBS and treated with 100 μg/mL RNase for 30 minutes, and then stained with 40 μg/mL PI.
Instrument	BD FACSCalibur
Software	FlowJo10.8.1
Cell population abundance	A minimum of 10,000 cells were analyzed per condition.
Gating strategy	FSC-A and FSC-H was used to identify single cells. DNA content were identified based on Propidium iodide staining.

- [x] Tick this box to confirm that a figure exemplifying the gating strategy is provided in the Supplementary Information.
